# Pitch profile across the cuticle of the scarab beetle *Cotinis* *mutabilis* determined by analysis of Mueller matrix measurements

**DOI:** 10.1098/rsos.181096

**Published:** 2018-12-05

**Authors:** Arturo Mendoza-Galván, Eloy Muñoz-Pineda, Kenneth Järrendahl, Hans Arwin

**Affiliations:** 1 Cinvestav-Unidad Querétaro, Libramiento Norponiente 2000, Querétaro 76230, Mexico; 2 Materials Optics, Department of Physics, Chemistry and Biology, Linköping University, SE-581 83 Linköping, Sweden

**Keywords:** structural colour, natural photonic structures, Bragg reflection, Bouligand structures

## Abstract

Helicoidal structures of lamellae of nanofibrils constitute the cuticle of some scarab beetles with iridescent metallic-like shine reflecting left-handed polarized light. The spectral and polarization properties of the reflected light depend on the pitch of the helicoidal structures, dispersion of effective refractive indices and thicknesses of layers in the cuticle. By modelling the outer exocuticle of the scarab beetle *Cotinis mutabilis* as a stack of continuously twisted biaxial slices of transparent materials, we extract optical and structural parameters by nonlinear regression analysis of variable-angle Mueller-matrix spectroscopic data. Inhomogeneities in the beetle cuticle produce depolarization with non-uniformity in cuticle thickness as the dominant effect. The pitch across the cuticle of *C*. *mutabilis* decreased with depth in a two-level profile from 380 to 335 nm and from 390 to 361 nm in greenish and reddish specimens, respectively, whereas in a yellowish specimen, the pitch decreased with depth in a three-level profile from 388 to 326 nm.

## 1. Introduction

**Figure 1.**
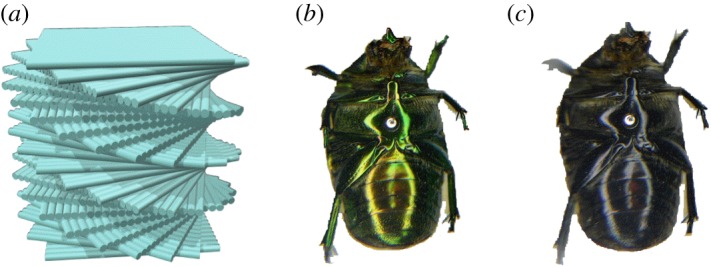
(*a*) Schematics of a Bouligand structure, (*b*,*c*) pictures of *C. mutabilis* taken in unpolarized light on and through a filter of right-handed circular polarization, respectively.

Many beetles exhibit brilliant colours caused by the interaction of light with structures in their exoskeletons [1,2]. More than a century ago, Michelson proposed that reflection of circularly polarized light from the scarab beetle *Plusiotis resplendens* (now *Chrysina resplendens*) was due to a ‘screw structure’ [3]. Subsequent studies on the cuticle of beetles exhibiting a reflection of circularly polarized light showed that they can be viewed as optical analogues to chiral nematic liquid crystals [4]. Indeed, it has been hypothesized that a pathway of cuticle morphogenesis may include a cholesteric liquid crystal phase [5,6]. Nowadays, comprehensive studies have proven that circularly polarized light reflection is common in many scarab beetles [7]. The ability to selectively reflect circularly polarized light is termed the circular Bragg phenomenon (or selective Bragg reflection) [8]. The current explanation of the selective Bragg reflection in beetles makes use of the so-called Bouligand structure [9], which is formed by the helicoidal arrangement of fibrous materials as illustrated in figure 1*a*. Thus, at normal incidence, selective Bragg reflection of the co-handed mode as the Bouligand structure is observed at wavelength *λ*_0_ = *n*_av_*Λ*, where *n*_av_ is the in-plane average refractive index and *Λ* the helix pitch (the distance for a full 360° rotation of fibrils). Natural helicoidal structures might appear in more complex arrangements [10,11].

Among the approaches that have been used to study the helicoidal structure and optical functions of the beetle cuticle are the reflection of unpolarized incident light [12–16], reflection of co-handed and cross-polarized circularly polarized light [17–19], and determination of cuticle Mueller matrices [20,21]. Often, features in the experimental spectra are explained by comparing with simulated data assuming predetermined structures like helicoids with single-valued pitch [19,20], helicoidal chiral slabs of two pitches in series [12,13], single-pitch structure with a jump in the orientation of fibrils [13,19] and chirped structures [16,21]. In some cases, electron microscopy has been employed to determine the pitch profile of the helicoidal structure [14–18]. Although valuable results have been reported in the references cited above, several details need further investigation. Among them, we identify four issues: (1) in most of those works, dispersion of refractive indices is ignored; (2) only in-plane anisotropy is considered because most of the measurements were performed at near-normal incidence (less than 25°); (3) the cuticle of beetles is treated as an ideal optical system, i.e. the reflected light is assumed to be totally polarized; (4) determination of the variation in pitch across the cuticle has not been assessed by optical means. In our research group, issues (1) and (2) have been addressed by a regression analysis of variable-angle Mueller-matrix spectroscopic ellipsometry data from the cuticle of the beetle *Cetonia aurata* [22]. Issue (3) was (partially) addressed for *C*. *aurata* by modelling the data as the incoherent superposition of contributions of areas with a different pitch, but the degree of polarization was not evaluated. In summary, given the diversity of species of beetles with cuticles as selective Bragg reflectors, the determination of their architectures and composition is crucial for a better understanding of their morphogenesis and functionality. In that sense, the application of the Mueller-matrix approach can contribute to achieve that objective, as it allows the determination of dispersion in the refractive indices, pitch profile of helicoids with a graded pitch and deviations from ideal optical systems (origin of light depolarization).

Regarding issue (4), we recently proposed that the cuticle of the scarab beetle *Cotinis mutabilis* (Gory and Percheron, 1833) might be comprised of a series of chiral slabs with different pitch values [23]. The latter structure was deduced from an analysis of interference oscillations in Mueller-matrix spectroscopic ellipsometry data in terms of the optical modes of wave propagation in a helicoidal structure. Subsequently, we demonstrated that scratching the cuticle of the beetle *C*. *mutabilis*, the spectral (colour) and polarization properties of the surface and inner zones differ [24]. Such differences were ascribed to different pitch values across the cuticle. However, implementation of a methodology addressing issues (1) to (4) is pending.

In this work, we systematically address issues (1) to (4) above by representing beetle cuticle with a model that includes a dispersion of refractive indices, in-plane and out-of-plane anisotropy, deviation of an ideal system and an analytic function describing twist variation of chitin-based fibrils to describe the pitch profile across the cuticle. We perform a nonlinear regression analysis of experimental Mueller-matrix data to determine the graded pitch profile, thicknesses and dispersion of refractive indices. Non-uniformity in thickness accounts for depolarization observed in the experimental Mueller matrix. To account for variations in the species, data from three specimens of different colours of *C*. *mutabilis* are analysed. The complexity of the system requires the incorporation of information found in our previous works for this species [23–25].

## 2. Basics of the Stokes–Mueller formalism

In this formalism, light beams are represented by Stokes vectors (**S**) and the sample is characterized by a 4 × 4 Mueller matrix **M** = {*M_ij_*}. The Stokes vectors have the components [26],

2.1 $$S=\left[\begin{array}{c} I\\ Q\\ U\\ V \end{array}\right],$$

where *I* = *I*_p_ + *I*_s_, accounts for the total irradiance. *Q* = *I*_p_ − *I*_s_ and *U* = *I*_+45°_ − *I*_−45°_ are irradiances describing linear polarization. Here *p* is parallel to and *s* is perpendicular to the plane of incidence and +45° and −45° are measured from the plane of incidence. The fourth component *V* = *I*_R_ − *I*_L_ accounts for circular polarization where *R* and *L* stand for irradiances of right- and left-handed, respectively. The incident (**S***_i_*) and reflected (**S***_r_*) light beams are related by,

2.2 $$S_{r}=MS_{i}.$$

Since the reflected beam **S**_*r*_ = [*I*,*Q*,*U*,*V*]^T^ (T meaning transpose) in general will be partially polarized, the degree of polarization *P* can be calculated from [26],

2.3 $$P=\frac{\sqrt{Q^{2}+U^{2}+V^{2}}}{I}.$$

In general, the polarized part of the reflected irradiance will be elliptically polarized with ellipticity (*e*) given by [26],

2.4 $$e=\tan\left(\frac{1}{2}\arcsin\frac{V}{\sqrt{Q^{2}+U^{2}+V^{2}}}\right),$$

where −1 ≤ *e* ≤ 1. The extrema correspond to left- and right-handed circular polarization, respectively, *e* = 0 is for linear polarization and other values of *e* are for elliptical polarization.

A unique capability of the Mueller-matrix approach is a quantification of the depolarization introduced by the sample. This is done by the depolarizance (*D*) which is an average measure of the depolarization produced by a system for all incident pure states, *D* is given by [27],

2.5 $$D=1-\left|\;{\left[\frac{1}{3}\left(\frac{tr(M^{T}M)}{M_{11}^{2}}-1\right)\right]}^{1/2}\right|\;.$$

where 0 ≤ *D* ≤ 1 and *tr* stands for trace. For non-depolarizing (ideal) system *D* = 0, whereas *D* = 1 is for an ideal depolarizer. In this work, normalized Mueller matrices (*m_ij_* = *M_ij_*/*M*_11_) and **S**_*i*_ (*I*_p_ *+* *I*_s_ = 1) are used.

## 3. Overview of the structure and Mueller-matrix data of beetle cuticle

### 3.1. Cuticle structure

The specimens of the beetle *Cotinis mutabilis* under study were collected at Querétaro, Mexico. Experimental details are specified in the electronic supplementary material. Figure 1*b* shows a picture taken in unpolarized light on a specimen of which the abdominal side exhibits a yellowish shiny metallic-like colour. Pictures of reddish and greenish specimens are shown in the electronic supplementary material, figure S1.

**Figure 2.**
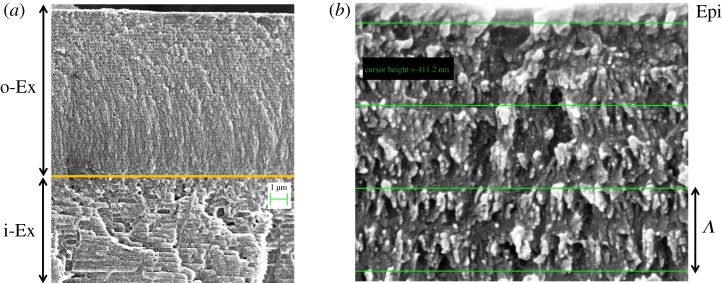
Cross section of the cuticle of *C. mutabilis* imaged by scanning electron microscopy: (*a*) outer exocuticle (o-Ex) and inner exocuticle (i-Ex) of a yellowish specimen; (*b*) near the surface region of a reddish specimen showing the epicuticle (Epi) and the fibrous arrangement comprising the Bouligand structure.

The cuticle of beetles is comprised of three regions identified by their microstructure and composition, which from top to bottom are epicuticle, exocuticle and endocuticle. Figure 2 shows scanning electron images of the cross section of the cuticle of two specimens of *C. mutabilis*. In figure 2*a*, two different regions are further distinguished in the exocuticle: the outer exocuticle and the tanned inner exocuticle whose thicknesses are about 9.5 and 7.2 µm, respectively. The endocuticle is located beneath the inner exocuticle. Figure 2*b* shows the near-to-the-surface region of a reddish specimen where the epicuticle can be resolved at the top with a thickness of about 100 nm. Most interesting is the fibrous structure of the outer exocuticle where grey and bright zones are distinguished. According to the scheme of the Bouligand structure in figure 1*a*, the bright spots imaged in figure 2*b* correspond to the tips of fibrils running perpendicular to the cut plane. On the other hand, fibrils running nearly parallel to the cut plane are imaged as the grey lamellae in figure 2*b*. The pitch corresponds to the distance between two lamellae as illustrated at the bottom of figure 2*b*, in this case about 411 nm according to the resolution of the lines drawn during image acquisition.

### 3.2. Experimental Mueller-matrix data

**Figure 3.**
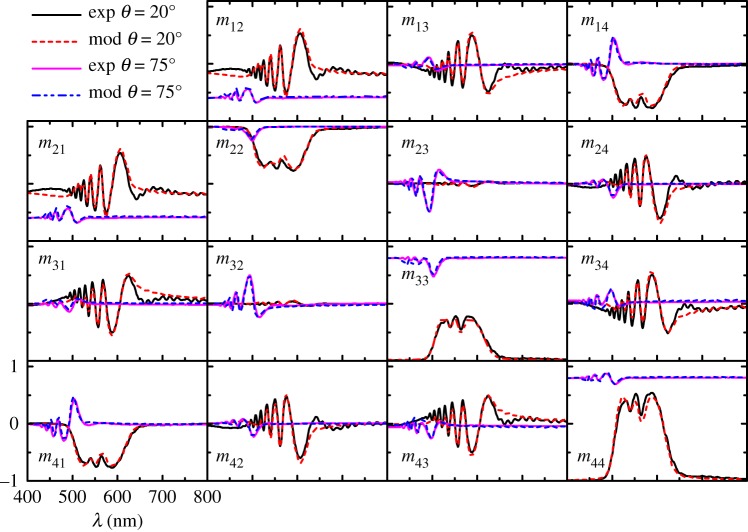
Experimental and best fit of Mueller matrices of a yellowish specimen for angles of incidence *θ* = 20° and 75°. Scales are shown on the lower left panel.

Figure 3 shows Mueller-matrix spectroscopic ellipsometry data measured at angles of incidence *θ* = 20° and 75° with a dual rotating compensator ellipsometer (RC2 system, J. A. Woollam Co., Inc.). Experimental details and full data measured are given in the electronic supplementary material, figures S2 and S3. The best fit data included in figure 3 were calculated with the model described below in §4. Spectral details and angle of incidence dependence were thoroughly discussed in our previous works [23,25] and are not repeated here. Only few key points are remarked in the next paragraph.

**Figure 4.**
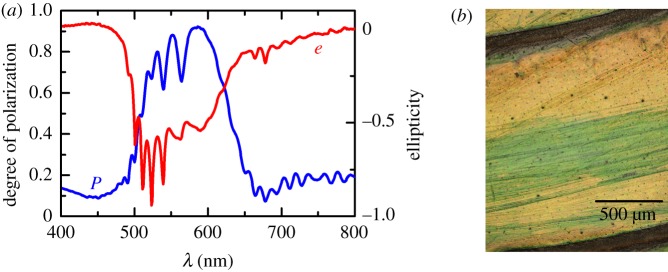
(*a*) Degree of polarization (*P*) and ellipticity (*e*) of light reflected on *C*. *mutabilis* for unpolarized incident light at *θ* = 20°. (*b*) Optical microscopy image of intact (yellowish) and scratched (green) zones in the cuticle of *C*. *mutabilis*.

Focusing on data measured at *θ* = 20°, negative values of *m*_41_ at wavelengths in the range of 485–650 nm define the band of selective reflection of left-handed polarized light. For unpolarized incident light, **S**_*i*_ = [1,0,0,0]^T^ equation (2.2) gives **S**_*r*_ = [1, *m*_21_, *m*_31_, *m*_41_]^T^, from which the degree of polarization and ellipticity shown in figure 4*a* were calculated with equations (2.3) and (2.4), respectively. The high degree of polarization and the left-hand character (*e* < 0) of the reflected beam explain the dark appearance of the beetle in figure 1*c*. Another salient feature in the experimental data of figure 3 is the presence of oscillations, which contain information on the propagation of electromagnetic waves analogous to chiral nematic liquid crystals as we previously have reported [23,24]. Furthermore, the outer exocuticle thickness could be estimated from the maxima and minima in the long-wavelength side of the selective Bragg reflection. Most interestingly, using a spectral analysis of oscillations, we previously anticipated that near the surface the pitch is larger than in deeper zones in the cuticle of *C*. *mutabilis* [23]. This result was corroborated by gently scratching the cuticle [24], as is shown in figure 4*b*, whereby a yellowish intact cuticle appears greenish after scratching. In the next section, the findings discussed so far are integrated into an optical model to determine the pitch profile and optical functions of the cuticle of *C*. *mutabilis* by regression analysis of Mueller-matrix data.

## 4. Modelling of beetle cuticle

### 4.1. Multilayer model

**Figure 5.**
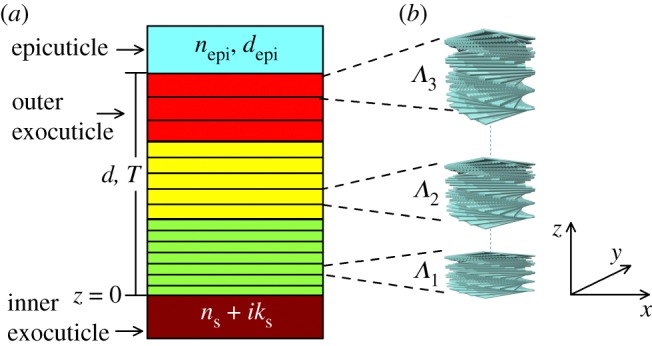
(*a*) Schematics of the multilayer structural model representing the epicuticle, outer exocuticle and the tanned inner exocuticle. (*b*) Illustration how twisted anisotropic slices comprise the helicoidal or Bouligand structures in the outer exocuticle.

Figure 5*a* shows a schematic of a structural model of the outermost part of the cuticle of *C*. *mutabilis* where selective Bragg reflection occurs. The epicuticle is represented as a non-absorbing isotropic layer of refractive index *n*_epi_ and thickness *d*_epi_. At the bottom, the tanned inner exocuticle acts as a substrate with complex refractive index *n*_s_ + *ik*_s_. The endocuticle is not considered in our model because it is not involved in the optical phenomena. The Bouligand structures are found in the outer exocuticle and are represented by three chiral slabs identified with a characteristic pitch *Λ_j_*. As was mentioned in §3.2 above, the pitch in the cuticle of *C*. *mutabilis* decreases with depth, as is schematically shown in figure 5*b* where *Λ*_3_ > *Λ*_2_ > *Λ*_1_.

### 4.2. Parametrization of graded pitch profile

Parametrization of the twisted fibrous arrangement of chitin–protein complexes of a single-pitch Bouligand structure of thickness *d* has been described in detail before [22] and is summarized in the electronic supplementary material. Modelling the cuticle as a cascade of chiral slabs with abrupt changes of the pitch is straightforward. However, considering cuticle morphogenesis as a continuous process, one expects a continuous variation of the twist of chitin fibrils lamellae across the cuticle producing a gradual change of the pitch. In the model, the helicoidal structure is represented by twisted anisotropic slices which are characterized by the principal components of the dielectric tensor diag[*ɛ*_1_,*ɛ*_2_,*ɛ*_3_] along the *x-*, *y*- and *z*-axes as shown in figure 5*b*. Alternatively, the principal components of the refractive indices (*n*_1_,*n*_2_,*n*_3_) can be used. The same composition across the outer exocuticle is assumed. The twist of anisotropic slices is parametrized as a variable azimuth angle (in degrees), which locates the orientation of *ɛ*_1_ as,

4.1 $$\phi (z)=\phi_{0}+360T\left(z/d-\sum_{\;j=1}^{2}a_{j}\ln\{1+\exp[\left(z-z_{0j}\right)/\left(db_{j}\right)]\}\right)\;,$$

where *z* is the position measured from the bottom, *d* is the outer exocuticle thickness, *T* is the number of full (360°) turns and *ϕ*_0_ the azimuth offset of *ɛ*_1_ with respect to the plane of incidence. The parameters *a_j_*, *z*_0*j*_ and *b_j_* are, respectively, the strength, position and broadening of the *j*-th change of pitch. Defining the cumulated number of periods as

4.2 $$N_{p}=\frac{\phi -\phi_{0}}{360},$$

the pitch profile is determined by the derivative,

4.3 $$\Lambda ={\left(\frac{dN_{p}}{dz}\right)}^{-1}\;.$$

The applicability of equation (4.1) to determine the pitch profile is demonstrated in the electronic supplementary material.

### 4.3. Outline of Mueller-matrix calculations

In this work, the calculations of Mueller matrices were performed with the CompleteEASE software (J. A. Woollam Co., Inc.). The model described in figure 5*a* is ideal in the sense that for any incident totally polarized beam, the reflected light is also totally polarized, that is, the model represents a non-depolarizing sample. Therefore, the 2 × 2 Jones matrix **J** = {*r_ij_*} of the entire multilayer can be calculated first. In the Jones formalism, **J** relates the p- and s- components of the incident (*E*_i_) and reflected (*E*_r_) electric field of the electromagnetic waves as [26],

4.4 $$\left[\begin{array}{c} E_{\mathrm{rp}}\\ E_{\mathrm{rs}} \end{array}\right]=\left[\begin{array}{cc} r_{\mathrm{pp}} & r_{\mathrm{ps}}\\ r_{\mathrm{sp}} & r_{\mathrm{ss}} \end{array}\right]\left[\begin{array}{c} E_{\mathrm{ip}}\\ E_{\mathrm{is}} \end{array}\right]\;.$$

Then, the Mueller matrix of the non-depolarizing optical system represented by the Jones matrix in equation (4.4) can be calculated with the standard procedure $M_{J}=T(J⊗J*)T^{-1}$ where ⊗ denotes the Kronecker product, the asterisk means complex conjugation and **T** is the matrix relating the Stokes and coherency vectors [26]. The components of the coherency vector are the elements of the coherency matrix in the Jones representation. Finally, to calculate the Mueller matrix of the model in figure 5*a*, it is necessary to specify the values of thicknesses (*d*,*d*_epi_), refractive indices (*n*_epi_,*n*_1_,*n*_2_,*n*_3_,*n*_s_ + *ik*_s_) and parameters in equation (4.1) that determine the pitch profile. The procedure to determine the initial values of these parameters is described in the electronic supplementary material.

### 4.4. Non-ideal cuticle

Mueller matrices of beetles do, however, depolarize polarized incident light [23,25]. Therefore, deviations from an ideal model must be included to consider this effect. In the present case, we assume non-uniformity in the thickness of the cuticle of *C*. *mutabilis* as the source of depolarization. Thus, Mueller matrices are calculated for nine thicknesses within the interval *d* − Δ*d* and *d* + *Δd*. The Gaussian weighted average of these Mueller matrices represents the incoherent superposition of light reflected from the non-uniform cuticle causing light depolarization.

## 5. Results and discussion

### 5.1. Fitted Mueller matrices

Figure 3 shows the best fit Mueller matrices of a yellowish specimen for *θ* = 20° and 75°. It can be noted that the model represents a very good description of the experimental data, apart from small deviations in *m*_12_, *m*_13_, *m*_24_ and *m*_34_ (as well as in their symmetric elements) in the range 600–700 nm. The full set of fitted variable-angle Mueller matrices is shown in the polar contour maps of electronic supplementary material, figure S2. Furthermore, experimental and best fit Mueller matrices of greenish and reddish specimens at selected angles of incidence are shown in electronic supplementary material, figures S4 and S5, respectively. The validity of the assumption on the origin of light depolarization produced by beetle cuticle, i.e. non-uniform thickness, is corroborated in the electronic supplementary material, figure S6. The deviation between experimental and data calculated in the range 600–700 nm in figure 3 is ascribed to inhomogeneities in pitch near the surface and can also be observed as an experimental depolarizance larger than the model calculated. Including such effects would increase model complexity leading to larger parameter correlation and take focus from the main model features.

Table 1 shows the structural parameters determined by the regression analysis of Mueller-matrix data of three specimens. It can be noted that the thickness of the epicuticle is very similar for the specimens analysed. However, the thickness of the outer exocuticle shows an appreciable variation between specimens from 9.2 to 6.6 µm. On the other hand, non-uniformity in thickness does not show an appreciable variation. The full set of fitted parameters and confident limits are shown in electronic supplementary material, table S1 and correlation matrices in electronic supplementary material, tables S2–S4.

**Table 1.** Epicuticle (*d*_epi_) and outer exocuticle (*d*) thickness, offset azimuth (*ϕ*_0_), number of turns (*T*) and non-uniformity in thickness (Δ*d*/*d*) determined for the cuticle of three specimens of the scarab beetle *C*. *mutabilis*.

	Specimen		
parameter	yellowish	greenish	reddish
*d*_epi_ (nm)	70 ± 1	87 ± 1	89 ± 1
*d* (μm)	9.244 ± 0.006	7.843 ± 0.007	6.573 ± 0.006
*ϕ*_0_ (°)	−50 ± 2	−31 ± 2	−34 ± 2
*T*	28.43 ± 0.03	23.40 ± 0.02	18.23 ± 0.01
Δ*d*/*d* (%)	2.60 ± 0.02	2.23 ± 0.03	2.50 ± 0.04

### 5.2. Pitch profile across the cuticle of *C*. *mutabilis*

**Figure 6.**
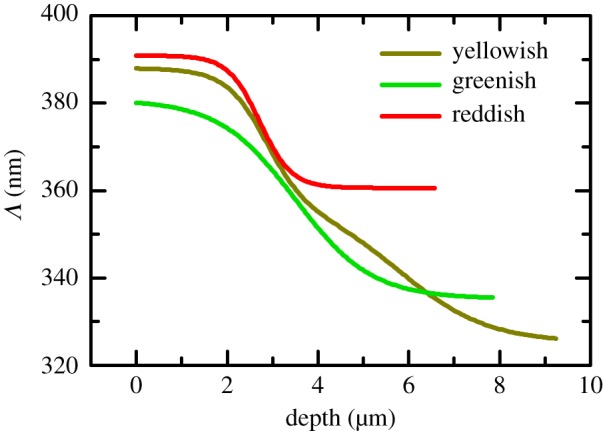
Pitch profile across the outer exocuticle of three specimens of *C*. *mutabilis* determined by regression analysis of Mueller matrices.

The pitch profiles determined across the cuticle of the three specimens are shown in figure 6. Near the cuticle surface of the yellowish specimen, the pitch is approximately 388 nm to a depth of about 2 µm. Then it steeply decreases to about 350 nm at 4 µm depth and slowly decreases to about 1.5 µm. At about 5.5 µm in depth, the pitch decreases faster to about 326 nm at the bottom of the outer exocuticle. In total, the three-level pitch decreases by about 62 nm across the cuticle. On the other hand, the pitch across the cuticle of the other specimens shows a two-level profile. For the greenish specimen, the change is from 380 to 335 nm at 3.6 µm whereas for the reddish specimen the pitch changes from 390 to 361 nm at about 2.7 µm. It should be noted that the pitch near the surface determined from the optical analysis for the reddish specimen is only 5% lower than the one estimated in the SEM image of figure 2*b*.

For comparison, the pitch values reported by other investigators for different species of beetles are discussed. Data from beetles showing similar colours to those analysed here were selected. In a green specimen of the scarab beetle *Gymnopleurus virens*, electron microscopy studies on the thorax revealed a pitch of 310 nm in the near surface region and in deeper regions, a pitch of 348 nm, whereas in a red specimen, a single-pitch structure of 384 nm was found [14]. Also, a double-pitch structure in the elytron of the scarab beetle *Plusiotis boucardi* was reported with a short pitch 310 nm overlaying a longer pitch 370 nm [13]. In the latter work, the pitch values were deduced from simulations of reflectance assuming the two-pitch structure. In another report, eight of 209 analysed *Lomaptera* beetles were identified as double-pitch structures with a variation between 310 and 390 nm [20].

### 5.3. Dispersion of effective refractive indices in the cuticle of *C*. *mutabilis*

**Figure 7.**
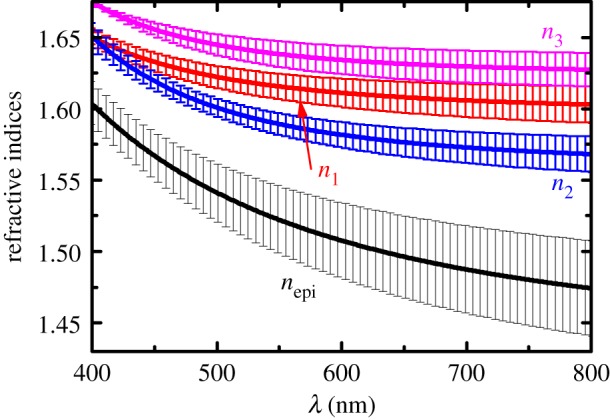
Effective refractive indices of epicuticle (*n*_epi_) and principal components (*n*_1_,*n*_2_,*n*_3_) of the anisotropic slices modelling the outer exocuticle of *C*. *mutabilis*. Averaged values (solid lines) and standard deviation bars were calculated from data of three specimens.

The epicuticle refractive index *n*_epi_ and the principal refractive indices (*n*_1_,*n*_2_,*n*_3_) of the outer exocuticle determined by regression analysis using Mueller-matrix data are shown in electronic supplementary material, figures S7 and S8. We observe that they differ among specimens but the average values of (*n*_1_,*n*_2_,*n*_3_) for the three specimens follow a trend as can be seen in figure 7, where for clarity only the spectral range near to selective Bragg reflection is shown. The dispersion observed in the effective refractive indices can be ascribed to the main constituents of beetle cuticle, chitin [28] and proteins [29], which both are absorbing in the ultraviolet range.

Clearly, *n*_3_ > *n*_1_ > *n*_2_ in most of the spectral range of figure 7. Since *n*_3_ > *n*_av_ = (*n*_1_ + *n*_2_)/2 the cuticle has a positive out-of-plane birefringence, Δ*n*_⊥_ = *n*_3_ − *n*_av_, in agreement with polarized optical microscopy studies in other species [8]. At wavelength 550 nm, we have *n*_av_ = 1.61, in-plane birefringence *Δn*_|__|_ = *n*_1_ − *n*_2_ = 0.0272, and Δ*n*_⊥_ = 0.0265, both decreasing as the wavelength decreases. The latter values of birefringence are one order of magnitude larger than what we earlier determined for chitin films [30]. Furthermore, the values of (*n*_1_,*n*_2_,*n*_3_) are higher than the refractive index reported for butterfly chitin [31] as can be seen in electronic supplementary material, figure S8. Therefore, the structural and chemical conformation of chitin–protein complexes might produce a strong influence on the optical properties of the cuticle. In works of other authors, dispersion of refractive indices in the cuticle of beetles showing selective Bragg reflection was not considered [13,14,20]. Additionally, the refractive indices determined in this work lie between the high and low refractive indices determined for the Bragg reflector in the cuticle of the jewel beetle *Chrysochroa fulgidissima* [32], as can be seen in electronic supplementary material, figure S8. Further studies are required to give a detailed explanation on the origin of anisotropy in the beetle cuticle. However, the results in figure 7 may help for a better understanding of the conformational arrangement of chitin–protein complexes.

The reliability of the refractive indices and birefringence obtained in this work is confirmed when they are compared to data from beetles with selective Bragg reflection reported by other authors. As was mentioned in the Introduction, in most of those reports dispersion in refractive index is often neglected. In the scarab beetle *G. virens* reported values are *n*_av_ = 1.61 and Δ*n* = 0.06 [14]; for the scarab beetle *P. boucardi* average values *n*_1_ = 1.70 and *n*_2_ = 1.58, that is *n*_av_ = 1.64 and Δ*n*_|__|_ = 0.12 [13]; for *Lomaptera* beetles in-plane complex refractive indices were reported for several species [20], averaging 1.484 + 0.0086*i* and 1.516 + 0.0086*i*, for the two in-plane components, in this case, *n*_av_ = 1.5 and Δ*n* = 0.032. As seen in electronic supplementary material, figure S2 (e.g. in *m*_41_), the selective reflection band shifts to shorter wavelengths and becomes narrower for increasing angles of incidence. The narrowing is due to smaller in-plane birefringence at shorter wavelengths as seen in figure 7. Finally, parameters for the epicuticle of other species have been reported and for the scarab beetle *P. boucardi*, a thickness of 0.75 µm and refractive index 1.41 were found [13], whereas a uniaxial epicuticle 0.5 µm thick with the same refractive indices as in the helicoidal exocuticle were considered for the scarab beetle *G. virens* [14].

## 6. Concluding remarks

Spectral variable-angle Mueller matrices measured on the cuticle of the scarab beetle *C*. *mutabilis* have been modelled as continuously twisted biaxial slices. The model provides a parametrized expression that describes the pitch profile across beetle exocuticle. Effective refractive indices with Cauchy dispersion are used to represent the materials comprising the cuticle. Parameters of three specimens exemplifying the colour variation found in beetle species were determined. It was found that the epicuticle is isotropic with a thickness ranging between 70 and 89 nm among specimens. At 550 nm, the central wavelength of the selective Bragg band, the in-plane and out-of-plane birefringence are 0.027 and 0.04, respectively, and both decrease as the wavelength decreases. The thickness of the outer exocuticle varies among specimens ranging from 6.6 to 9.2 µm. The pitch across the cuticle gradually varies with depth from 388 to 326 nm (−62 nm) in a yellowish specimen, from 380 to 335 nm (−45 nm) in the greenish specimen whereas for the reddish specimen the pitch changes from 390 to 361 nm (−29 nm).

## Supplementary Material

Supplementary information

## Permission to carry out fieldwork

The specimens of the beetle *Cotinis mutabilis* were collected at Querétaro, Mexico. No special collecting permit or ‘Animal Care Protocol’ was required at the time. Arturo Mendoza Galván, a Mexican citizen and faculty researcher at Cinvestav, was responsible for collecting the specimens.

## Data accessibility

Data, experimental and analysis procedures, as well as additional figures and tables supporting the results, are provided as electronic supplementary material.
