# Supplementary material for: Pitch profile across the cuticle of the scarab beetle Cotinis mutabilis determined by analysis of Mueller matrix measurements
Source: R Soc Open Sci. 2018 Dec 5;5(12):181096. doi: 10.1098/rsos.181096 (PMC6304155; doi:10.1098/rsos.181096)
Supplement: Supplementary information [file rsos181096supp1.pdf]

Supplementary Information

Pitch profile across the cuticle of the scarab beetle *Cotinis mutabilis* determined by analysis of Mueller matrix measurements

Arturo Mendoza-Galván,[1,2,*] Eloy Muñoz-Pineda,[1] Kenneth Järrendahl,[2] and Hans Arwin[2]

[1]*Cinvestav-Unidad Querétaro, Libramiento Norponiente 2000, Fracc. Real de Juriquilla, Querétaro, 76230 Mexico*

[2]*Materials Optics, Department of Physics, Chemistry and Biology, Linköping University, SE-581 83 Linköping, Sweden*

*Corresponding author: amendoza@cinvestav.mx; arturo.mendoza@liu.se

Contents

Experimental

Overview of the experimental Mueller matrix data

Model details

Supplementary figures:

Figure S1. Pictures of specimens of *Cotinis mutabilis.*

Figure S2. Polar contour maps of experimental and fitted Mueller matrices.

Figure S3. Experimental and best-fit of Mueller matrices of a yellowish specimen.

Figure S4. Experimental and best-fit of Mueller matrices of a greenish specimen.

Figure S5. Experimental and best-fit of Mueller matrices of a reddish specimen.

Figure S6. Experimental and model calculated depolarizance of Mueller matrices.

Figure S7. Epicuticle refractive indices of three specimens.

Figure S8. Principal components of refractive indices of the anisotropic slices representing the helicoidal structure in the outer exocuticle of three specimens.

References

Experimental

The specimens of the beetle *C. mutabilis* under study were collected at Querétaro, Mexico, No special collecting permit or "Animal Care Protocol" was required at the time. Arturo Mendoza Galván, a Mexican citizen and researcher at Cinvestav, was responsible for collecting the specimens. Figure S1 shows pictures of the three specimens analysed. As can be seen, the abdominal side presents a shiny metallic-like colour (greenish, yellowish, or reddish). For the study, areas as flat as possible were selected from the segments in the abdomen and a small piece of about 2×3 mm^2 was cut using a sharp knife. The piece of the cuticle was mounted on a glass slide with double-sided tape for the Mueller-matrix spectroscopic ellipsometry measurements performed with a dual rotating compensator ellipsometer (RC2 system, J. A. Woollam Co., Inc.). The measurements were performed at angles of incidence (θ) between 20 and 75° in steps of 5° in the wavelength (λ) range 245 to 1000 nm. Since the cuticle is slightly curved, focusing probes were used to achieve a beam spot with size below 100 μm. Non-linear regression analysis to fit model-generated data to experimental data was performed with the CompleteEASE software (J. A. Woollam Co., Inc.) which provides best-fit parameters and 90% confidence intervals. More details about the instrument and modelling can be found in references [1-3].

The electron micrographs were taken with a scanning electron microscope LEO 1550 Gemini. For these studies, the samples were mounted on double-side copper tape in such a way that the cross-section was exposed: The samples were coated with a thin platinum layer of approximately 2 nm to obtain a conductive surface.

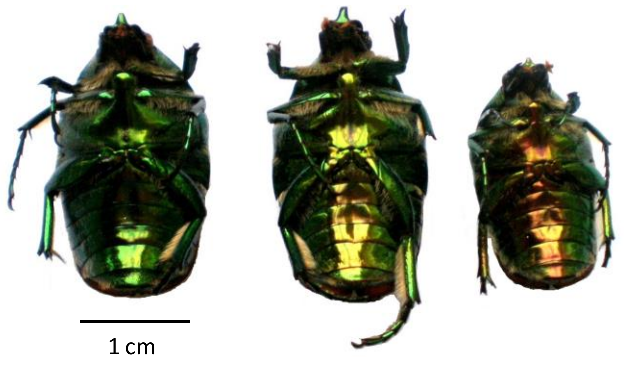

Figure S1. Pictures of greenish, yellowish, and reddish specimens of *C. mutabilis*.

Overview of the experimental Mueller matrix data

Figure S2 (left) shows polar contour maps of the experimental Mueller matrices of *C. mutabilis* measured at angles of incidence between 20 and 75° (the polar angle) in the wavelength range 245 to 1000 nm (the radial axis). The features of the variable-angle Mueller matrices are similar to those previously reported for other specimens and exhaustively described before [2,3]. In particular, we emphasize four major features: i) for non-polarized incident light with $\mathbf{S}_i=[1,0,0,0]^T$ the reflected beam represented by $\mathbf{S}_r=[1,m_{21},m_{31},m_{41}]^T$ is left-handed polarized ($m_{41}<0$) at low angles of incidence in the narrow spectral range of 485 to 650 nm, consistent with figure 1(c); ii) symmetries between the elements describe a chiral system [2], namely, $m_{12}=m_{21}$, $m_{13}=-m_{31}$, $m_{14}=m_{41}$, $m_{23}=-m_{32}$, $m_{24}=m_{42}$, and $m_{34}=-m_{43}$, leaving nine symmetry-independent elements in **M**; iii) the pseudo-isotropic character outside the Bragg reflection band, i.e. the Mueller matrix is nearly block-diagonal; iv) the shift of the selective Bragg reflection to shorter wavelengths at increasing angles of incidence characteristic of an all-dielectric system. The model-calculated Mueller matrices on the right of figure S2, gives a very good description of all features in the experimental data.

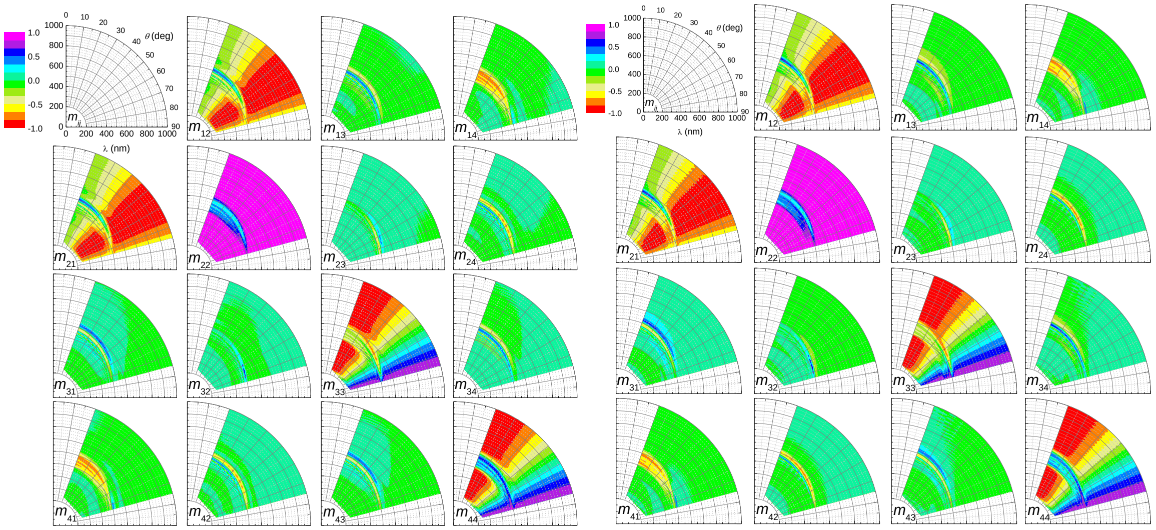

Figure S2. Experimental (left) and model-calculated (right) polar contour map of Mueller-matrices of a yellowish specimen of *C. mutabilis* at angles of incidence between 20 and 75°. The scale is shown in the upper-left corner.

Model details

To calculate the Mueller matrix of the model in figure 5(a) it is necessary specify the values of various parameters: thicknesses (d,d_{epi}), refractive indices (n_{epi},n_1,n_2,n_3,n_s+ik_s) and parameters in equation (9) that determine the pitch profile. The procedure to assign initial values of model parameters is described below.

Thicknesses. As was described in previous reports [3,4] and summarized in section 2.2, the thickness of the outer exocuticle can be estimated from a spectral analysis of maxima and minima of oscillations in m_{21} at θ=20° for wavelengths longer than 650 nm (outside the selective Bragg reflection band). We found d=9.8 μm for the yellowish specimen analysed in this work. Previously reported outer exocuticle thicknesses for other specimens estimated with the same procedure were 6.5 µm and 8.5 µm. For non-linear regression analysis the number of anisotropic slices should be large enough to describe a continuous variation of the azimuth in equation (6) of the main text. In this work we have used 500 anisotropic slices which fulfil thus the requirement of continuity [1]. For the epicuticle, we set d_{epi}=80 nm according to electron microscopy studies of specimens of *C. mutabilis* [2,3].

Refractive indices. Because the Mueller-matrix data of *C. mutabilis* are characterized by strong oscillations, we consider transparent materials comprising the epicuticle and outer exocuticle. Therefore, n_{epi} and the three refractive indices (n_1,n_2,n_3) corresponding to the dielectric function tensor diag[ε_1,ε_2,ε_3]= diag[n_1^2,n_2^2,n_3^2] were represented with the Cauchy dispersion relation,

$$n_j = A_j + B_j \big/ \lambda^2 + C_j \big/ \lambda^4 \ , \tag{S1}$$

where A_j, B_j, and C_j are fitting parameters defined as ≥0. By using wavelengths expressed in units of μm in equation (S1), the initial values of (A_j, B_j, C_j) of the various refractive indices in the cuticle were n_{epi}=(1.42, 0.009, 0.003), n_1=(1.5, 0.015, 0.001), n_2=(1.47, 0.015, 0.001), and n_3=n_1. The initial choice n_3=n_1, was appropriate to obtain the correct sign of m_{31} mostly at large angles of incidence. For the tanned inner exocuticle we used the same complex refractive index (N_s =n_s+ik_s) used before for the beetle *C. aurata* [1].

Parameterization of a single chiral stack. In this case, the twist of anisotropic slices representing the helicoidal structure is parameterized as a variable azimuth angle (in degrees), which locates the orientation of ε_1 [1],

$$\phi(z) = \phi_0 + 360Tz/d\ , \tag{S2}$$

where z is the position measured from the bottom, d the thickness of the structure, T is the number of turns, and ϕ_0 the azimuth offset of ε_1 with respect to the plane of incidence. Defining the cumulated number of periods as,

$$N_\mathrm{p} = (\phi - \phi_0)/360\ , \tag{S3}$$

we get, $z=\Lambda N_\mathrm{p}$ where $\Lambda=d/T$ is the pitch of the chiral layer, i.e. the inverse slope of the N_p vs. z curve. This can be generalized by the derivative,

$$\Lambda = \left(\frac{dN_\mathrm{p}}{dz}\right)^{-1}\ . \tag{S4}$$

Graded pitch profile. An appropriate function that describes the gradual change of the pitch is given by,

$$\Lambda(z) = \Lambda_1 + \sum_{j=1}^{2} \frac{\Delta\Lambda_j}{1+\exp\left[-\left(z-z_{0j}\right)/\gamma_j\right]}\ . \tag{S5}$$

where Λ_1, $\Lambda_1+\Delta\Lambda_1$ ($=\Lambda_2$), and $\Lambda_2+\Delta\Lambda_2$ ($=\Lambda_3$) are the pitches near the bottom (z=0), intermediate, and near the top of the cuticle ($z=d$), respectively; z_{0j} is the centre of the j-th transition and γ_j the broadening. However, equation (S5) cannot been implemented directly in the analysis software. Therefore, the challenge was to find an appropriate analytical expression for $\phi(z)$. This resulted in our ansatz,

$$\phi(z) = \phi_0 + 360T\left(z/d - \sum_{j=1}^{2} a_j \ln\left\{1+\exp\left[\left(z-z_{0j}\right)/\left(db_j\right)\right]\right\}\right), \tag{S6}$$

where a_j, z_{0j}, and b_j are, respectively, the strength, position, and broadening of the j-th change of pitch. Note that in equation (S6) a_j and b_j are dimensionless parameters. From equations (S3), (S4) and (S6) we get the pitch distribution as a function of z as,

$$\Lambda(z) = \frac{d/T}{1-\sum_{j=1}^{2}\left(a_j/b_j\right)\left\{1+\exp\left[-\left(z-z_{0j}\right)/\left(db_j\right)\right]\right\}^{-1}}\ . \tag{S7}$$

Furthermore, for values such that a_i/b_j <<1 (i.e. for sharp transitions), equation (S7) can be written in first approximation as,

$$\Lambda(z) \approx \frac{d}{T} + \sum_{j=1}^{2} \frac{a_j d/(b_j T)}{1+\exp[-(z-z_{0j})/(db_j)]}. \quad \text{(S8)}$$

Number of pitches. The number of minima in the experimental spectrum of m_{41} at θ=20°was used to define the number of pitch levels in the outer exocuticle. For data from the yellowish specimen, figure 3, three minima in m_{41} are located at λ_j =521, 557, and 590 nm (j=1,2,3). At oblique incidence, selective Bragg reflection occurs at $\lambda_j=n_{av}\Lambda_j\cos\theta_t$ where θ_t is the angle of propagation of electromagnetic waves inside the helicoidal structure and it is determined from Snell's law, $\sin\theta=n_{av}\sin\theta_t$ [5], taking $n_{av}=(n_1+n_2)/2=1.55$ as a suitable value for the in-plane average refractive index [4]. Thus, the estimated initial pitch levels were Λ_j= 343, 368 and 390 nm. In the case of the greenish and reddish specimens two-pitch structures were considered.

Pitch profile parameters. The values of Λ_j were used to determine the initial values of other parameters in equation (S6). For the yellowish specimen, the number of turns T=28.6 was calculated from the deepest pitch $\Lambda_1=d/T$. The strength (a_j) and width (b_j) of the transitions from one pitch to other were determined by trial and error to obtain the appropriate steps $\Lambda_1\rightarrow\Lambda_2\rightarrow\Lambda_3$. We found $a_1=a_2=0.003$ and $b_1=b_2=0.04$ to be appropriate. It should be noted that these initial values fulfil the condition imposed to obtain equation (S8). Finally, the position of each step was chosen by considering that the number of oscillations in m_{21} at the short-wavelength side of the selective Bragg reflection depend on the depth of pitch transitions [4]. From those considerations, the values $z_{01}=0.33d$ and $z_{02}=0.67d$ were appropriate. A similar procedure was followed to set values to initial parameters to analyse data from the greenish and reddish specimens.

Azimuth offset. The initial value of ϕ_0 was determined by simulations noting that this parameter mostly affects the phase of the oscillations in m_{21} (as well as in m_{31}, m_{42}, m_{42}, m_{43}, and other symmetry-related elements) within the selective Bragg reflection.

Non-ideal cuticle. Simulations with such (ideal) model produce spectra with strong oscillations in m_{21} (and other elements) at the band of selective Bragg reflection. However, Mueller matrices of beetles depolarize incident polarized light [2,3] and, in the present case, we assume a non-uniform thickness of the cuticle of *C. mutabilis* as the source of depolarization. An appropriate damping of the oscillations was obtained by introducing an initial 2% non-uniformity in outer exocuticle thickness.

Non-linear regression analysis

Non-linear regression analysis was performed with the CompleteEASE software (J. A. Woollam Co., Inc.) which provides best-fit parameters and 90% confidence intervals. In this procedure, the mean squared error (MSE) is minimized by using the Levenberg-Marquardt algorithm [1],

$$MSE = \frac{1000}{L - M_{\mathrm{p}}} \sum_{l=1}^{L} \sum_{i,j=1}^{4} \left[\left(m_{ij,l}^{\mathrm{exp}} - m_{ij,l}^{\mathrm{mod}} (\mathbf{x}) \right)^2 \right] \qquad \text{(S9)}$$

where $L=L_\lambda L_\theta$ is the product of the number of wavelengths (L_λ) and angles of incidence (L_θ) data points, $m_{ij,l}^{\mathrm{exp}}$ and $m_{ij,l}^{\mathrm{mod}}$ are the elements of the experimental and model Mueller matrix, respectively, and M_{p} is the number of parameters in the parameter vector $\mathbf{x}$ which has as components the thicknesses (d,d_{epi}), Cauchy coefficients A_j, B_j, and C_j in equation (S1) of refractive indices (n_{epi},n_1,n_2,n_3), and parameters in equation (S6) that determine the pitch profile.

Fitted Mueller matrices

As can be noticed, the fitted variable-angle Mueller matrix shown in the polar contour maps of figure S2 gives a very good description of the experimental data. Figures S3 to S5 show experimental and fitted Mueller matrices, at selected angles of incidence of yellowish, greenish, and reddish specimens of *C. mutabilis*. Again, the model offers a very good description.

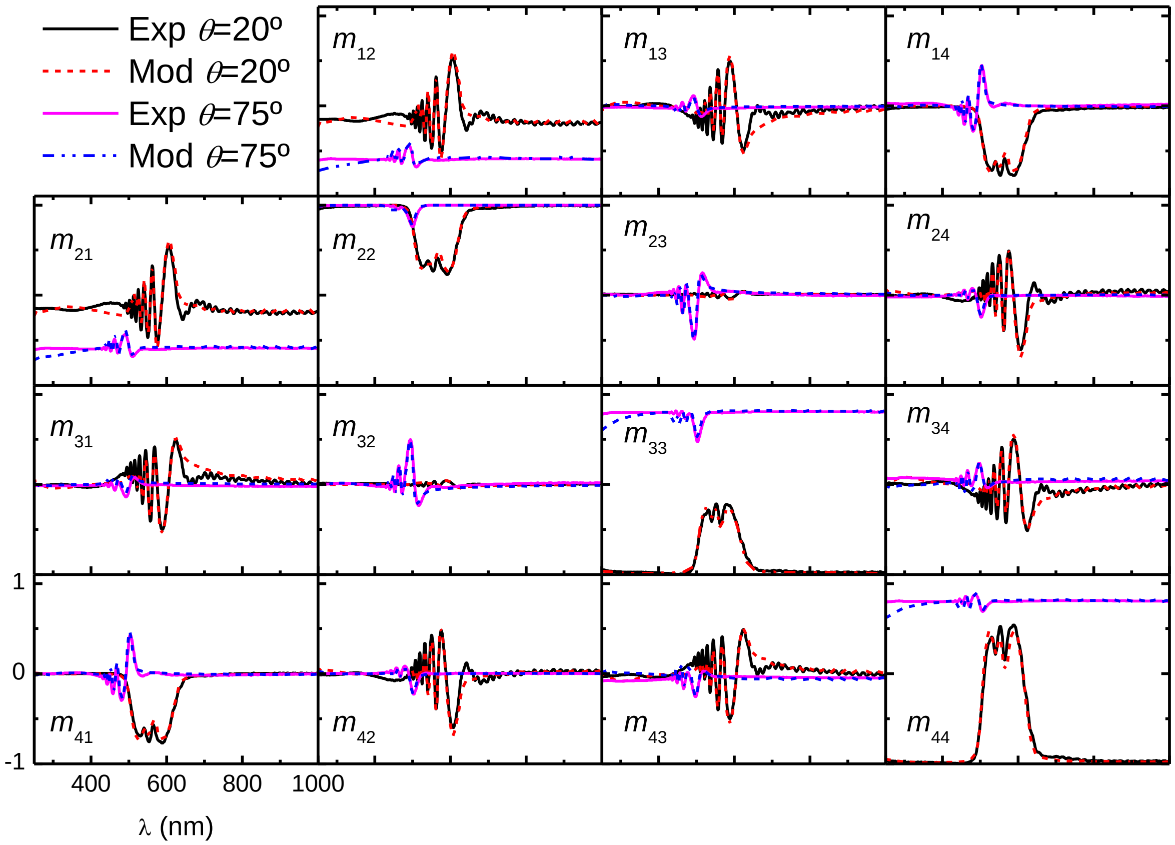

Figure S3. Experimental and best-fit of Mueller matrices of the yellowish specimen for angles of incidence θ=20 and 75°. Scales are shown on the lower-left panel.

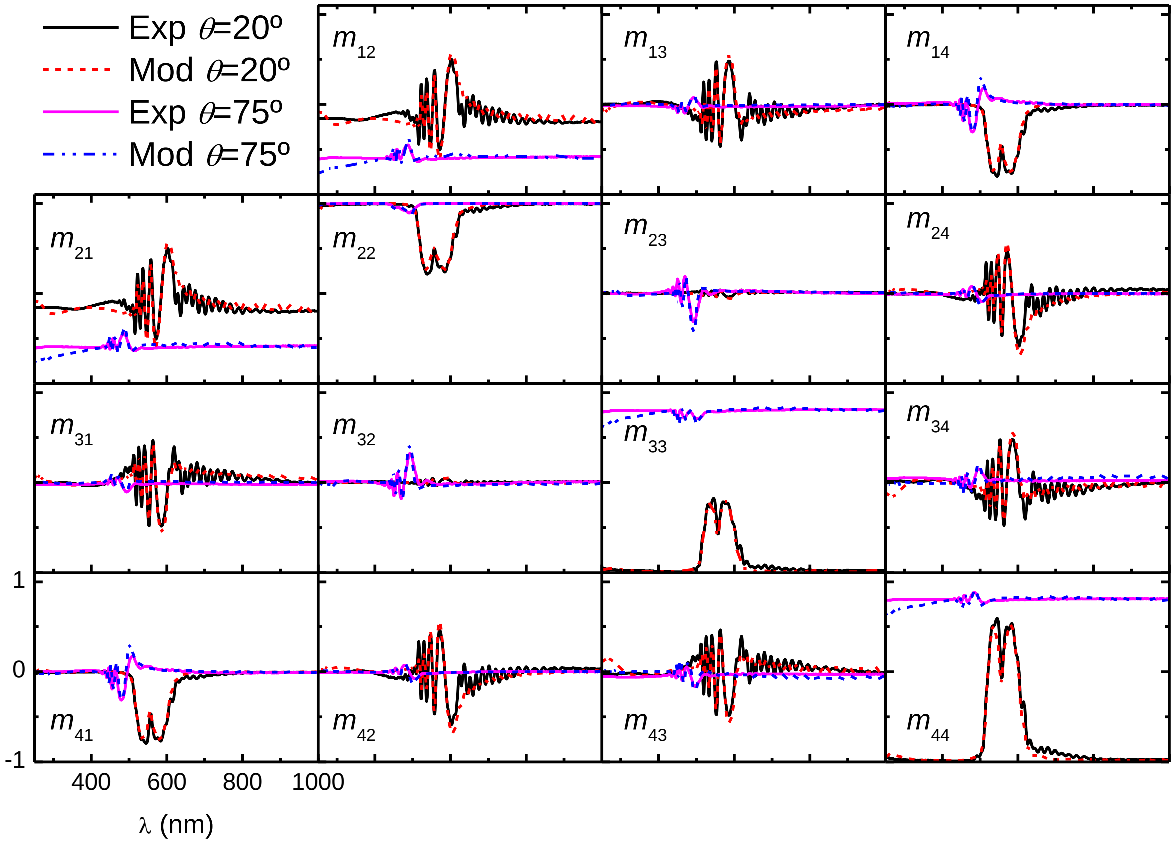

Figure S4. Experimental and best-fit of Mueller matrices of a greenish specimen for angles of incidence θ=20 and 75°. Scales are shown on the lower-left panel.

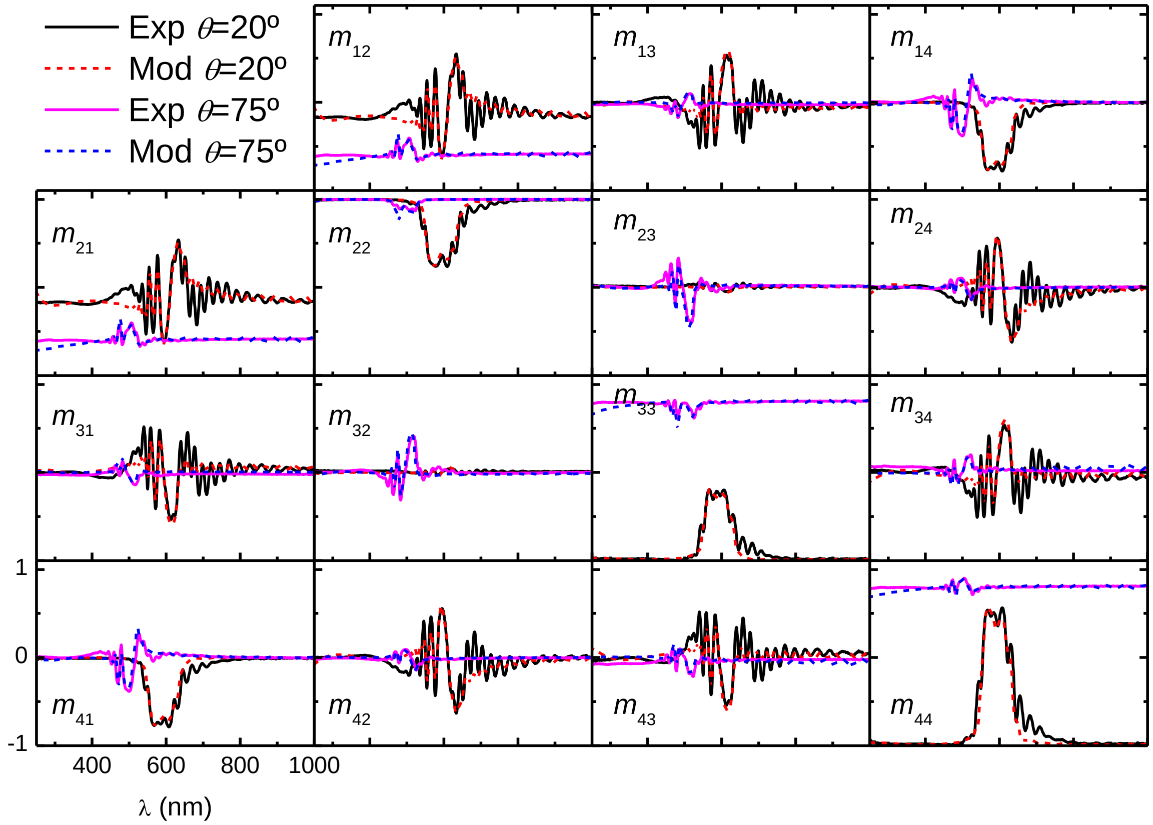

Figure S5. Experimental and best-fit of Mueller matrices of a reddish specimen for angles of incidence θ=20 and 75°. Scales are shown on the lower-left panel.

Depolarization caused by the cuticle of *C. mutabilis*

Because non-uniformity in thickness was introduced as the source of depolarization, it is necessary to determine the validity of this assumption. An appropriate way is to quantify the depolarizance (*D*) of the experimental and best-fit Mueller matrices according to [6],

$$D = 1 - P_{\Delta} = 1 - \left[\frac{1}{3}\left(\frac{tr\left(\mathbf{M}^{T}\mathbf{M}\right)}{M_{11}^{2}} - 1\right)\right]^{1/2}, \tag{S10}$$

where P_{Δ} is the degree of polarimetric purity (also called depolarization index) and *tr* stands for trace. Thus, *D* is an average measure of the depolarization produced by a system for all incident pure states. Figure S6 shows the depolarizance of the experimental and best-fit model Mueller matrices at angles of incidence θ=20 and 75°. In a model without non-uniformity in *d*, *D* would be zero. However, by fitting non-uniformity in thickness, a significant improvement in modelling of depolarization is achieved. For data from the

yellowish specimen, figure S6(a), the largest values of D for θ=20° appear in the wavelength range 490 to 580 nm, where the light beam probes deeper into the cuticle. The larger experimental depolarizance than the model-calculated at about 630 nm is ascribed to inhomogeneities in pitch near the surface. For a larger angle of incidence, D decreases because the cuticle behaves as a dielectric reflector [2,3]. The latter reason also explains the low values of D outside of the selective Bragg reflection, where a high degree of linear polarization has been reported [2,3]. A similar behaviour is found for data from the greenish and reddish specimens, figures S6(b) and S6(c), respectively.

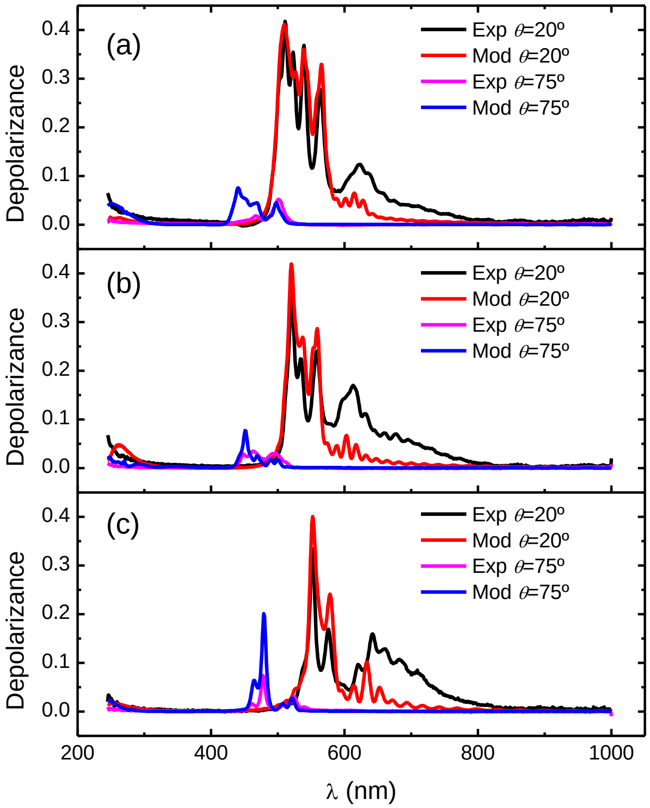

Figure S6. Experimental and model depolarizance of Mueller matrices of three specimens of *C. mutabilis* for selected angles of incidence: (a) yellowish, (b) greenish, and (c) reddish.

Effective refractive indices

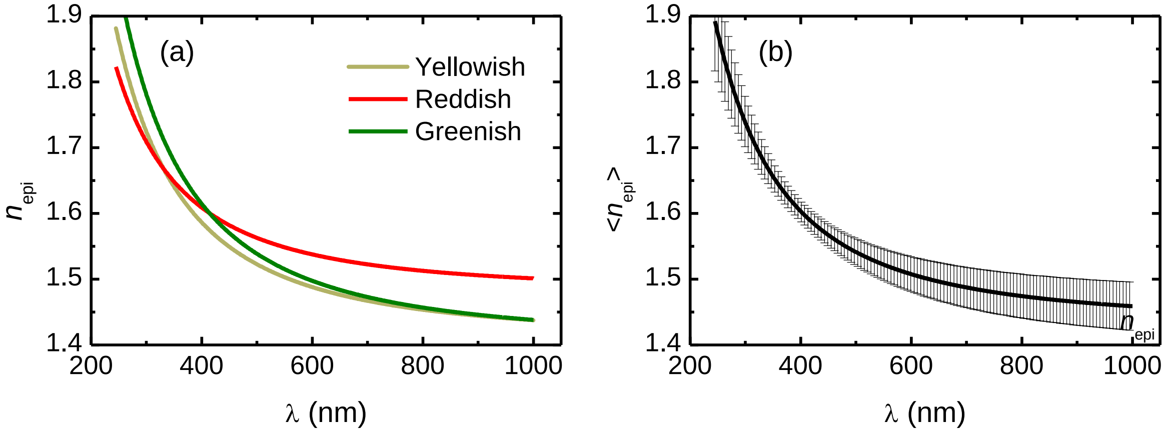

Figure S7. (a) Effective refractive indices of the epicuticle (n_{epi}) of yellowish, reddish, and greenish specimens of *C. mutabilis*. (b) Average and standard deviation.

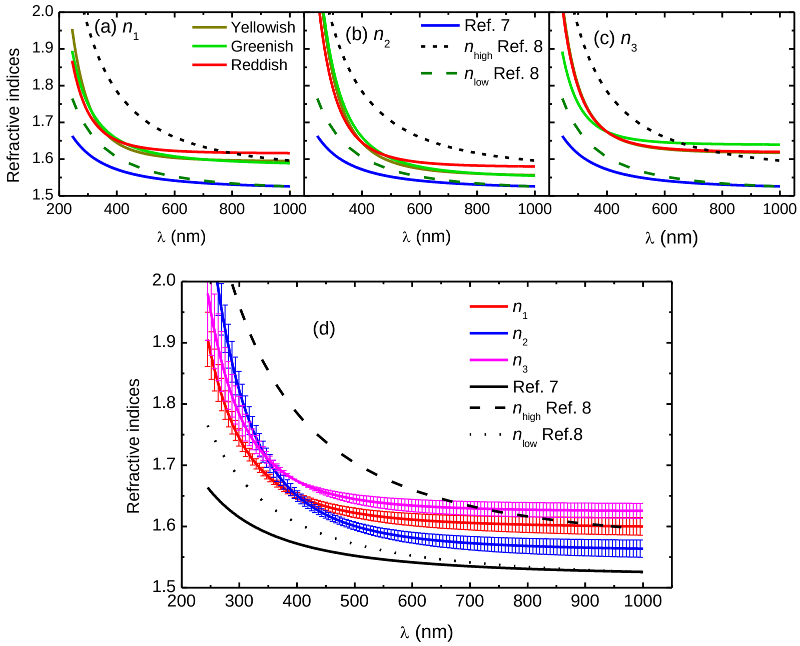

Figure S8. Effective principal components of refractive indices (a) n_1, (b) n_2, and (c) n_3, of the anisotropic slices modelling the outer exocuticle of yellowish, greenish, and reddish specimens of *C. mutabilis*. (d) Average values and standard deviations. For comparison, data for butterfly chitin [7] $n=1.517+8.8\times10^3/\lambda^2$ and low- and high- refractive indices determined for the Bragg reflector in the cuticle of the jewel beetle *Chrysochroa fulgidissima* [8] $n_{high}=1.56+3.6\times10^4/\lambda^2$ and $n_{low}=1.51+1.53\times10^4/\lambda^2$ are also shown.

Table S1. Values and confidence limits of fitting parameters for the three specimens.

	Yellowish	Greenish	Reddish
d_{epi} (nm)	70 ± 1	87 ± 1	89 ± 1
d (nm)	9244 ± 6	7843 ± 7	6573 ± 6
ϕ_0 (°)	-50 ± 2	-31 ± 2	-34 ± 2
T	28.43 ± 0.03	23.4 ± 0.02	18.23 ± 0.01
$\Delta d/d$ (%)	2.60 ± 0.02	2.23 ± 0.03	2.50 ± 0.04
A_{epi}	1.409 ± 0.006	1.405 ± 0.008	1.481 ± 0.007
B_{epi}	0.028 ± 0.002	0.033 ± 0.003	0.021 ± 0.003
C_{epi}	0*	6E-05 ± 0.0002	0*
A_1	1.594 ± 0.002	1.581 ± 0.002	1.615 ± 0.002
B_1	0*	0.0078 ± 0.0005	0*
C_1	0.00130 ± 3E-05	0.00066 ± 3E-05	0.00091 ± 4E-05
A_2	1.552 ± 0.002	1.546 ± 0.002	1.578 ± 0.002
B_2	0.0024 ± 0.0003	0.0070 ± 0.0004	0*
C_2	0.00201 ± 2E-05	0.00181 ± 3E-05	0.00172 ± 4E-05
A_3	1.616 ± 0.006	1.639 ± 0.009	1.618 ± 0.009
B_3	0*	0*	0*
C_3	0.0015 ± 0.0001	0.0009 ± 0.0002	0.0014 ± 0.0002
a_1	0.0095 ± 0.0004	0.0128 ± 0.0004	0.0041 ± 0.0002
z_{01}/d	0.362 ± 0.009	0.540 ± 0.003	0.582 ± 0.002
b_1	0.105 ± 0.002	0.107 ± 0.003	0.053 ± 0.003
a_2	0.0033 ± 0.0003	-	-
z_{02}/d	0.687 ± 0.003	-	-
b_2	0.0468 ± 0.003	-	-

*These parameters attained value zero and therefore they were fixed for the final regression.

Table S2. Upper diagonal elements of the symmetric correlation matrix of fitting parameters for the yellowish specimen.

	d_{epi}	A_{epi}	B_{epi}	A_1	C_1	A_2	B_2	C_2	A_3	C_3	ϕ_0	T	a_1	z_{01}	b_1	a_2	z_{02}	b_2	d	Δd
d_{epi}	1.000	0.453	-0.468	0.148	-0.068	0.210	-0.143	0.172	0.125	0.089	0.011	-0.113	-0.014	0.021	-0.033	-0.056	0.009	-0.066	-0.298	-0.017
A_{epi}		1.000	-0.855	0.434	-0.168	0.215	0.011	0.096	0.020	0.155	0.055	-0.001	0.142	0.043	0.157	-0.061	0.024	-0.043	-0.445	-0.022
B_{epi}			1.000	-0.245	0.144	-0.141	0.019	-0.093	-0.076	-0.100	-0.050	0.042	-0.075	-0.039	-0.074	0.054	-0.029	0.047	0.284	0.014
A_1				1.000	-0.311	0.775	-0.179	0.278	0.085	0.236	0.096	-0.052	0.066	0.037	0.118	0.031	-0.036	0.063	-0.934	-0.056
C_1					1.000	-0.398	0.499	-0.656	-0.142	0.015	-0.135	0.020	-0.023	0.009	-0.054	-0.021	0.044	-0.035	0.198	-0.001
A_2						1.000	-0.727	0.701	0.202	0.125	0.158	-0.203	0.018	0.125	0.008	-0.041	0.079	-0.015	-0.749	0.026
B_2							1.000	-0.929	-0.219	0.068	-0.211	0.253	-0.004	-0.164	0.040	0.093	-0.132	0.076	0.179	-0.097
C_2								1.000	0.235	-0.069	0.277	-0.228	0.021	0.143	0.000	-0.068	0.079	-0.047	-0.286	0.078
A_3									1.000	-0.499	0.055	0.002	0.050	0.021	0.034	-0.039	0.051	-0.033	-0.148	0.056
C_3										1.000	0.052	-0.049	0.001	0.022	0.018	0.012	-0.023	0.018	-0.242	-0.017
ϕ_0											1.000	-0.231	0.189	0.126	0.234	-0.139	0.083	-0.116	-0.229	-0.085
T												1.000	0.081	-0.724	0.264	0.334	-0.366	0.267	0.141	-0.107
a_1													1.000	0.580	0.895	-0.743	0.647	-0.660	-0.061	-0.171
z_{01}														1.000	0.298	-0.787	0.770	-0.674	-0.097	-0.010
b_1															1.000	-0.418	0.288	-0.344	-0.092	-0.168
a_2																1.000	-0.860	0.978	-0.001	0.084
z_{02}																	1.000	-0.799	0.020	-0.091
b_2																		1.000	-0.031	0.069
d																			1.000	0.034
Δd																				1.000

Table S3. Upper diagonal elements of the symmetric correlation matrix of fitting parameters for the greenish specimen.

	d_{epi}	A_{epi}	B_{epi}	C_{epi}	A_1	B_1	C_1	A_2	B_2	C_2	A_3	C_3	ϕ_0	T	a_1	z_{01}	b_1	d	Δd
d_{epi}	1.000	0.017	0.176	-0.373	0.084	0.024	-0.078	0.168	-0.130	0.167	0.054	0.212	-0.063	-0.182	-0.087	-0.080	-0.075	-0.285	-0.013
A_{epi}		1.000	-0.911	0.761	0.216	-0.061	-0.015	0.056	0.100	-0.021	0.076	0.035	-0.019	0.035	0.069	-0.014	0.059	-0.238	-0.008
B_{epi}			1.000	-0.938	-0.083	0.029	0.030	0.023	-0.095	0.035	-0.105	0.072	0.005	-0.068	-0.095	-0.060	-0.080	0.053	0.009
C_{epi}				1.000	0.018	-0.000	-0.049	-0.056	0.089	-0.040	0.088	-0.148	-0.005	0.109	0.114	0.092	0.097	0.045	-0.006
A_1					1.000	-0.796	0.669	0.828	-0.475	0.354	0.447	0.059	-0.014	0.100	0.029	-0.298	0.040	-0.810	-0.070
B_1						1.000	-0.930	-0.554	0.493	-0.289	-0.468	0.106	0.011	-0.176	-0.059	0.358	-0.067	0.340	0.051
C_1							1.000	0.390	-0.303	0.041	0.391	-0.128	-0.026	0.171	0.046	-0.313	0.051	-0.235	-0.028
A_2								1.000	-0.797	0.695	0.433	0.050	0.031	0.042	0.062	-0.147	0.072	-0.775	-0.045
B_2									1.000	-0.918	-0.382	0.094	-0.109	-0.024	-0.074	0.105	-0.084	0.301	0.018
C_2										1.000	0.317	-0.102	0.182	-0.039	0.049	-0.062	0.058	-0.299	-0.027
A_3											1.000	-0.471	-0.009	0.124	0.107	-0.163	0.112	-0.309	0.013
C_3												1.000	0.056	-0.117	-0.073	0.021	-0.070	-0.204	-0.028
ϕ_0													1.000	-0.495	-0.166	0.092	-0.133	-0.168	-0.027
T														1.000	0.827	-0.540	0.800	0.085	-0.001
a_1															1.000	-0.204	0.995	0.028	-0.014
z_{01}																1.000	-0.215	0.138	0.022
b_1																	1.000	0.021	-0.020
d																		1.000	0.059
Δd																			1.000

Table S4. Upper diagonal elements of the symmetric correlation matrix of fitting parameters for the reddish specimen.

	d_{epi}	A_{epi}	B_{epi}	A_1	C_1	A_2	C_2	A_3	C_3	ϕ_0	T	a_1	z_{01}	b_1	d	Δd
d_{epi}	1.000	0.496	-0.572	0.114	0.023	0.099	0.054	-0.008	0.182	0.008	-0.415	-0.077	-0.010	-0.067	-0.339	0.002
A_{epi}		1.000	-0.845	0.323	-0.132	0.138	0.248	-0.089	0.190	0.060	-0.248	-0.016	-0.077	-0.012	-0.367	0.004
B_{epi}			1.000	-0.147	0.103	-0.060	-0.213	0.031	-0.182	-0.036	0.262	0.040	0.015	0.036	0.252	0.001
A_1				1.000	-0.182	0.919	0.146	0.182	0.065	0.122	-0.157	0.032	0.014	0.044	-0.905	-0.071
C_1					1.000	0.038	-0.461	-0.022	-0.030	0.062	-0.058	-0.032	0.075	-0.039	0.021	0.026
A_2						1.000	-0.113	0.237	0.033	0.065	-0.103	0.018	0.070	0.019	-0.874	-0.062
C_2							1.000	-0.008	0.021	0.130	-0.098	0.010	-0.044	0.018	-0.129	-0.026
A_3								1.000	-0.687	-0.036	0.018	0.040	0.028	0.045	-0. 197	0.034
C_3									1.000	0.064	-0.127	-0.035	0.019	-0.034	-0.103	-0.002
ϕ_0										1.000	-0.713	-0.141	0.011	-0.103	-0.367	0.025
T											1.000	0.469	-0.351	0.410	0.377	0.017
a_1												1.000	-0.010	0.993	0.008	-0.018
z_{01}													1.000	-0.020	-0.030	-0.105
b_1														1.000	-0.002	-0.021
d															1.000	0.050
Δd																1.000

References

1. Arwin H, Berlind T, Johs B, Järrendahl K. 2013 Cuticle structure of the scarab beetle *Cetonia aurata* analyzed by regression analysis of Mueller-matrix ellipsometric data. *Opt. Express* **21**, 22645-22656. (doi:10.1364/OE.21.022645)
2. Muñoz-Pineda E, Järrendahl K, Arwin H, Mendoza-Galván A. 2014 Symmetries and relationships between elements of the Mueller matrix spectra of the cuticle of the beetle *Cotinis mutabilis*. *Thin Solid Films* **571**, 660-665. (http://dx.doi.org/10.1016/j.tsf.2013.11.144)
3. Mendoza-Galván A, Muñoz-Pineda E, Järrendahl K, Arwin H, 2014 Evidence for a dispersion relation of optical modes in the cuticle of the scarab beetle *Cotinis mutabilis*. *Opt. Mater. Express* **4**, 2484-2496. (http://dx.doi.org/10.1364/OME.4.002484)
4. Mendoza-Galván A, Järrendahl K, Arwin H. 2017 Exposing different in-depth pitches in the cuticle of the scarab beetle *Cotinis mutabilis*. *Mater. Today: Proceed.* **4**, 4969-4978. (https://doi.org/10.1016/j.matpr.2017.04.103)
5. Takezoe H, Ouchi Y, Hara M, Fukuda A, Kuze E. 1983 Experimental studies of reflection spectra in monodomain cholesteric liquid crystal cells: total reflection, subsidiary oscillation and its beat or swell structure. *Jap. J. Appl. Phys.* **22**, 1080-1091. (http://dx.doi.org/10.1143/JJAP.22.1080)
6. Gil JJ. 2007 Polarimetric characterization of light and media. Eur. Phys. *J. Appl. Phys.* **40**, 1-47. (https://doi.org/10.1051/epjap:2007153)
7. Leertouwer HL, Wilts BD, Stavenga DG. 2011. Refractive index and dispersion of butterfly chitin and bird keratin measured by polarizing interference microscopy. *Opt. Express* **19**, 24061-24066. (https://doi.org/10.1364/OE.19.024061)
8. Yoshioka S, Kinoshita S. 2011. Direct determination of the refractive index of natural multilayer systems. *Phys. Rev. E* **83**, 051917. (https://journals.aps.org/pre/abstract/10.1103/PhysRevE.83.051917)
